# The Impact of Syphilis Screening among Female Sex Workers in China: A Modelling Study

**DOI:** 10.1371/journal.pone.0055622

**Published:** 2013-01-30

**Authors:** Kate M. Mitchell, Andrew P. Cox, David Mabey, Joseph D. Tucker, Rosanna W. Peeling, Peter Vickerman

**Affiliations:** 1 Department of Global Health and Development, London School of Hygiene and Tropical Medicine, London, United Kingdom; 2 Department of Infectious Disease Epidemiology, London School of Hygiene and Tropical Medicine, London, United Kingdom; 3 Department of Clinical Research, London School of Hygiene and Tropical Medicine, London, United Kingdom; 4 School of Medicine, University of North Carolina, Chapel Hill, North Carolina, United States of America; Yale School of Public Health, United States of America

## Abstract

**Background:**

In China, female sex workers (FSWs) are at high risk of syphilis infection, but are hard to reach for interventions. Point-of-care testing introduces opportunities for expanding syphilis control measures. Modelling is used to estimate the impact of using rapid tests to screen FSWs for syphilis. In other settings, modelling has predicted large rebounds in infectious syphilis following screening, which may undermine any impact achieved.

**Methods:**

A deterministic syphilis transmission model among FSWs and clients was fitted to data from Yunnan Province (FSW syphilis prevalence = 7.5%), and used to estimate the impact of rapid syphilis testing and treatment for FSWs. Impact projections were compared for different model structures that included risk heterogeneity amongst FSWs, incoming syphilis infections amongst new FSWs and clients and re-infection from FSWs' regular non-commercial partners. The rebound in syphilis prevalence after screening ceased was explored.

**Results:**

All model structures suggest yearly syphilis screening could substantially reduce (by 72–88%) syphilis prevalence amongst FSWs in this setting over five years. However, incoming syphilis infections amongst new FSWs and clients or re-infections from regular non-commercial partners of FSWs can considerably reduce (>30%) the proportion of infections averted. Including heterogeneity in risk amongst FSWs had little effect upon the proportion of infections averted. In this setting, the rebound in syphilis prevalence after screening ceased is predicted to be slight, but it could be large in high prevalence settings.

**Conclusions:**

Rapid test screening could dramatically reduce syphilis prevalence amongst hard-to-reach groups, but strategies to reduce re-infection from regular non-commercial partners are needed to maximise impact.

## Introduction

China has a large commercial sex industry with approximately 10 million female sex workers (FSWs) [1], who are hard to reach using conventional health outreach [2]. Commercial sex is mainly but not exclusively establishment based; in recent studies, the majority of FSWs (70–85%) report recruiting clients through establishments including karaoke clubs, beauty salons, night clubs, saunas, massage parlours, bars and hotels, while a much smaller percentage (15–30%) report recruiting clients on the street or by other means [3]–[5]. Syphilis is common in China [6], with the prevalence among FSWs varying from 0.4–43% [7]–[9].

Currently, syphilis diagnosis requires two laboratory tests (the rapid plasma reagin (RPR) test and confirmatory *Treponema pallidum* particle agglutination (TPPA) test) which need specialized equipment, making them difficult to perform rapidly and accurately in the field [10], [11]. This leads to delays between testing and treatment, and individuals not returning for treatment [12], [13]. There is interest in using new rapid diagnostic tests to diagnose and treat at the point of care, so expanding syphilis screening and treatment [13]. Several rapid tests are available, which detect treponemal antibody [14]. These tests require relatively little training or equipment, do not require a cold chain or electricity, and can be used on serum or plasma, or on whole blood obtained from a finger prick [14], making them ideal for use ‘on site’ with hard-to-reach groups. Rapid test sensitivity ranges from 64–100% and specificity from 95–100% [15]. However, since individuals usually retain treponemal antibodies for life once infected, even if infection is treated and cleared, these tests cannot distinguish between current and past infection [14]. This can lead to overtreatment of those who have been previously successfully treated [13]. Although there are few risks associated with overtreatment [16], there is concern that repeated intramuscular injections associated with treatment may deter people from retesting in future [13], and unnecessary treatment is expected to incur extra costs without giving any benefits.

Previous modelling studies have considered syphilis transmission in the general population [17]–[19], the transmission of other sexually transmitted infections (STIs) between FSWs and their clients [20], [21] (including the impact of treatment and rapid tests [22]–[25]), the effect of risk heterogeneity on STI epidemic dynamics [26], and the effect of ongoing partnerships on the impact of chlamydia screening [27]. To the best of our knowledge, no existing modelling studies have considered the implications for the impact of FSW-targeted STI treatment interventions of incorporating heterogeneity within a FSW population, or STI transmission between FSWs and their longer-term regular non-commercial partners.

Previous modelling analyses have suggested that wide-scale syphilis treatment can result in a subsequent substantial rebound in cases, where the prevalence of infectious syphilis rises far above levels seen in the absence of any treatment [18], [28]. It is therefore important to assess the likelihood and magnitude of a rebound when assessing the impact of a syphilis screening and treatment intervention.

Kaiyuan City in Yunnan province, China, has a large commercial sex industry and 7.5% syphilis prevalence among FSWs [4]. This analysis uses modelling to explore the impact of screening FSWs in Kaiyuan City for syphilis using existing rapid tests. The likelihood and magnitude of a post-intervention rebound was assessed. The impact projections incorporate uncertainty in the model parameters, and consider the importance of risk heterogeneity, incoming syphilis infections amongst new FSWs and clients and re-infection from the FSWs' regular non-commercial sex partners. We also evaluate the level of overtreatment that is likely to occur amongst those who have been previously treated and cured. These are expected to account for the large majority of over treatments since test specificity is high (98–100% in evaluations carried out in China [15]).

## Methods

### Model description

A deterministic dynamic model of syphilis transmission [17] between FSWs and their commercial clients was developed, with FSWs stratified into high- and low-risk groups, and a single group of clients mixing proportionately with the two FSW groups. An index j is used to represent the different population groups-low-risk FSWs (j = 1), high-risk FSWs (j = 2) or clients (j = 3), and index k represents the different stages of infection (k = 1–6). A more complex model including regular non-commercial partners of FSWs in a pairwise structure was also developed.

#### Transitions between infection states

**Figure 1:**
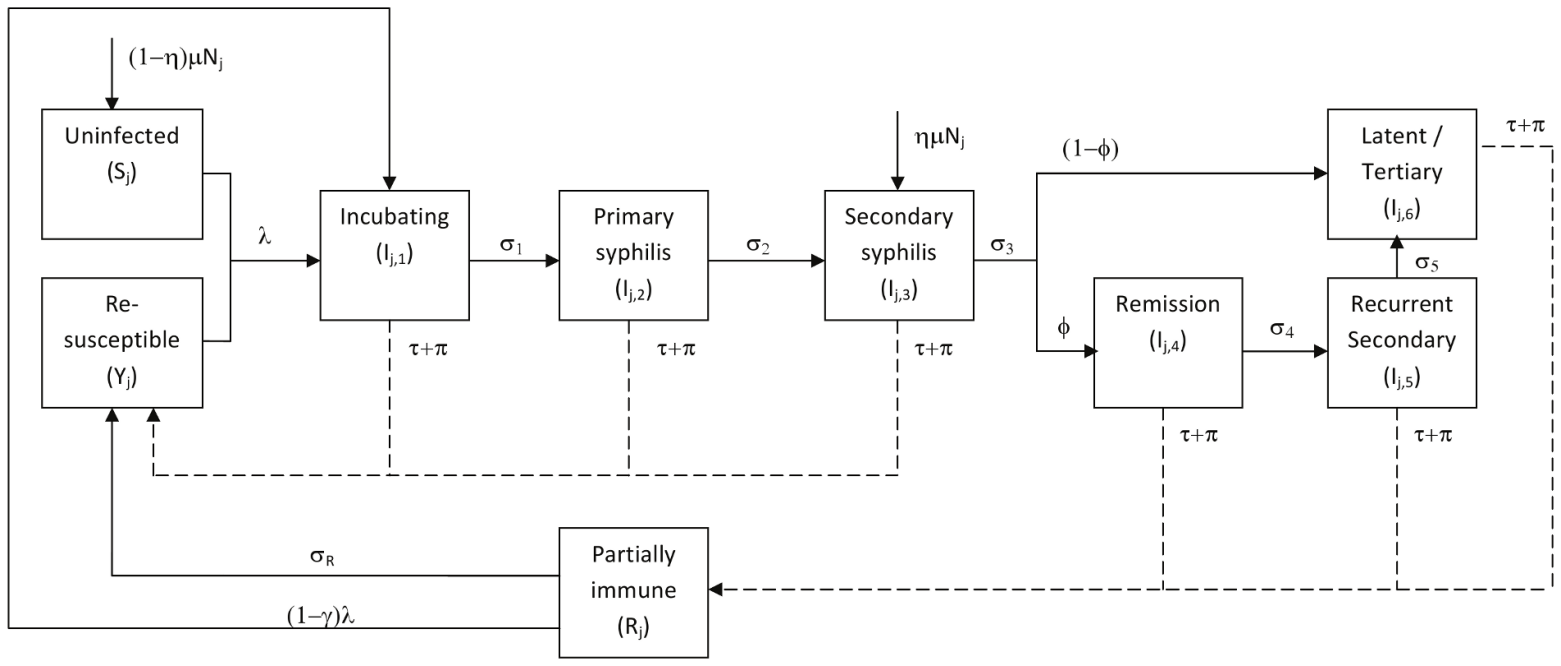
Model structure: syphilis stages and transitions. Schematic diagram showing different stages of infection for infected FSWs, clients and regular partners, and transitions between different stages. Leaving rates are not shown (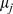 leaving every compartment). Solid lines show normal transitions in the natural history, dashed lines show transitions due to treatment.

Figure 1 shows the transitions between infection states. Susceptible individuals (S_j_) become infected at a per capita rate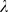, which is a function of the proportion of commercial partners who are infected (which varies over time), the number of different commercial partners per year, the likelihood of transmission per partnership, the relative infectiousness of the different infection stages and condom use. Infected individuals progress through an incubating state (I_j,1_, of average duration 1/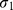 weeks) followed by the primary and secondary infection stages (I_j,2_ and I_j,3_, which last 1/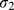 and 1/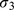 weeks, respectively). Following this, a proportion (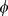) enter a period of remission (I_j,4_) before experiencing recurrent secondary infection (I_j,5_) followed by a latent/tertiary stage (I_j,6_) (with I_j,_ and I_j,5_ lasting 1/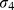 and 1/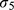 weeks respectively). The remainder (1−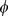)enter the latent/tertiary phase (I_j,6_) directly. The latent/tertiary phase is lifelong in the absence of treatment. Those in the primary and secondary stages (I_j,2_ and I_j,3_), and a proportion (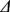) of those experiencing recurrent secondary infection (I_j,5_), are infectious for syphilis. All infected individuals (I_j,k = 1–6_) can be treated and cured of infection by background treatment (at per-capita rate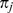) or screening and treatment (at per-capita rate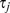). It is assumed that test sensitivity does not vary by infection stage. Following treatment, individuals in the incubating, primary, and secondary phases of infection (I_j,k = 1–3_) enter the re-susceptible class (Y_j_), while those in other infection stages (I_j,k = 4–6_) enter the partially immune class (R_j_), where they remain for 1/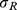 weeks before entering the re-susceptible class (Y_j_), consistent with the observation that immunity to syphilis is partial, temporary and dependent on infection duration [29]. Individuals in the partially immune state can become infected at a rate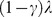, where 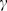
is the degree of immune protection. Individuals in the re-susceptible class are infected at the same rate, and follow the same natural history after re-infection as those never previously infected. FSW and client group sizes remain constant over time, with those leaving each group (at a rate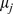) being replaced by new individuals. A fraction (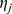) of new FSWs, clients and regular non-commercial partners of FSWs are assumed to be already infected with syphilis and enter the secondary syphilis stage (I_j,3_), while the remainder (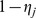) enter the susceptible (S) compartment. Model equations are presented in Text S1.

#### Model structures used

Four model variants were used to explore the effect of structural uncertainty on the impact of syphilis screening. Model 1 assumed no FSW behavioural heterogeneity and no incoming infections amongst new FSWs or clients (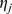 = 0). Model 2 assumed heterogeneity in the FSW population with high- and low-risk FSWs differing in their frequency of commercial sex, duration of sex work and condom use. Model 3 assumed the same risk heterogeneity, and additionally assumed that some incoming FSWs and clients were infected with syphilis (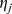
>0). Model 4 incorporated these same factors but also included the regular non-commercial partners of FSWs, with syphilis transmission occurring between the FSWs and these regular partners. Regular partners were included using pairwise partnership modelling [30], [31], with separate compartments for FSWs that were single, had an uninfected regular non-commercial partner, or had an infected regular partner in each stage of infection (see Text S1 and Figure S1).

### Parameter uncertainty ranges

Syphilis transmission and natural history model parameters were obtained from the literature [17]. FSW behavioural parameters were obtained from Kaiyuan City [4]. Parameters for the low- and high-risk groups came from FSWs practising commercial sex in low-risk venues (saunas, karaoke clubs, hotels and night clubs) or high-risk ones (streetwalkers, temporary sublets and beauty salons) [4]. These were combined to produce weighted averages for model 1. Client behavioural parameters were assigned wide ranges based on studies elsewhere in China [32], [33] or other countries [34], [35]. Data from Kaiyuan City [4] and elsewhere in China [36], [37] were used to produce point estimates for the behavioural parameters relating to regular non-commercial partners. The percentage of FSWs with a regular partner, and levels of condom use with these partners were those reported by FSWs in Kaiyuan, while estimates for the duration of regular partnerships and frequency of sex within these partnerships were taken from general population studies of long-term couples. The syphilis prevalence amongst premarital individuals in China was used to estimate the prevalence of syphilis in new clients and FSWs [8]. Table 1 gives the values and ranges used for each parameter.

**Table 1:** Model parameter values with uncertainty ranges.

Description	Symbol	Estimated Value	Range	Source
Syphilis epidemiological and transmission related parameters				
Transmission probability per sex act	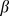	0.3	0.092–0.627	Estimates from prospective controlled trials reviewed in Garnett *et al*. (1997) [17], [43], [44]
Duration of syphilis infection by stage in weeks				
Incubating	1/ 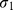	3.57	3.00–4.00	Data from experimental and natural human infections [17], [45]
Primary	1/ 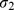	-	2.66–6.57	Data from experimental and natural human infections [17], [46], [47]
Secondary	1/ 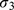	15.43	4.29–52.14	From study of untreated patients in Oslo [17], [48], [49]
Remission	1/ 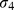	26	4.29–52.14	Oslo study of untreated patients noted that 23.6% of patients suffered a relapse, and 90% of these occurred within 1 year [17], [49]
Recurrent secondary	1/ 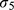			Assumed same as secondary [17], [48]
Latent Tertiary	1/ 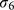	Lifelong (i.e. 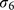 = 0)	-	Assumed lifelong, as in previous modelling studies [17], [18]
Proportion entering recurrent secondary (via remission)	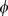	0.236	0.21–0.26	Oslo study of untreated patients found 23.6% of 1,035 patients developed recurrent secondary infection [48]
Duration of immunity in weeks	1/ 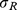	-	0–1300	No data available. Previous modelling by Grassly *et al.* (2005) [19] suggests duration of immunity is between 5–25 years. Very wide range of 0–25 years used here
Degree of partial immune protection	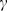	-	0–1	No data available so wide range used
Proportion infectious in recurrent secondary phase	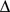	0.20	0.1–0.3	Depends on the prevalence of wet genital lesions in the recurrent secondary stage. Mandell *et al*. 2005 [50] estimate this as 20%, and a wide range of+/−50% is used
Efficacy of condoms in protecting against transmission of syphilis	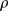	-	0.365–0.85	A longitudinal study [51] suggests relative reduction in risk of 36.5% (lower bound). Condoms have been found to be 85% effective in preventing HIV transmission [52](upper bound)
Behavioural parameters for female sex workers and their clients, and for regular partnerships of FSW				
FSW population size	N_1_+N_2_	1,000	-	Fixed number used, split into two groups as described below
Client population size	N_3_			Calculated by balancing demand and supply of commercial sex using equation N_3_ = (C_1_N_1_+C_2_N_2_)/C_3_
Proportion of FSW who have no regular partner	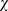	0.48	-	Fixed-data from FSW in Kaiyuan City study [4]
Proportion of FSW in low risk group	N_2_/(N_1_+N_2_)	0.621	0.4–0.7	Proportions in Kaiyuan City study from low risk venues [4]; wide range added to account for possible non-representative sampling
Duration of participation in commercial sex, in weeks				
FSW low risk group	1/ 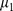	176.8	72.6–291.5	Linear interpolation used to estimate the median and inter-quartile range from categorical current duration data in Kaiyuan City study [4] doubled to give total duration instead of current duration (AP Cox, unpublished data)
FSW high risk group	1/ 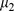	219.3	85.4–445.2	
Clients Overall	1/ 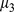	-	390–1291.2	No data from China available so data from elsewhere [34], [35] gives current duration of 48–150 months. This was doubled to give absolute duration of up to 300 months.
Duration of regular partnerships in weeks	1/ 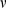	520	-	Fixed estimate; assumes average partnership duration of 10 years which is similar to China studies [36], [37]
Frequency of commercial sex per week				
FSW low risk group	C_l_	2.3	0.75–3.77	Weighted (by sample size in each venue) median obtained across venues defined as high or low risk by Wang *et al*. [4]+/−66% added to each to get ranges as this is the size of the overall interquartile range
FSW high risk group	C_h_	4.7	1.57–7.83	
Clients Overall	C_cl_	-	0.07–0.3	Two estimates from Yunnan [32] and Sichuan [33] provinces
Frequency of sex with regular partners per week	C_r_	1.3	-	Fixed estimate; estimates from different settings in China suggest 1–2 per week [36], [37]
Consistency of condom use				
Low risk FSW with clients	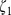	88.2%	84.9–91.0%	Data from Kaiyuan City [4]
High risk FSW with clients	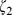	76.0%	70.5–80.9%	
FSW with regular partners	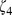	30%	-	Fixed estimate; 16% of FSW report always using condoms with regular partners in Kaiyuan City [4]-average usage assumed to be higher.
Background rate of treatment per week				
FSW	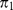	-	0.19–0.96%	No data-assumed 10–50% are treated per year.
Clients	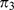	-	0.19–0.96%	
Regular partners	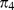	0	-	No data-assumed to be 0 in simulations shown or alternatively same as 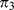
Syphilis prevalence data used for model fitting				
FSW low risk group		6.1%	4.1–8.7%	Data from Kaiyuan City [4] (RPR and TPPA positive)
FSW high risk group		9.7%	6.5–13.8%	
FSW Overall		7.5%	5.7–9.6%	
Clients Overall		2.4%	1–7.4%	No data from Kaiyuan city but 2.4% (1–4.6%) elsewhere in Yunnan Province [32] (RPR and TPPA); 5.3% (3.7–7.4%) in study from Sichuan province [38]
New FSWs and clients	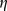	0.66%	0.31–1.43%	Median and IQR for prevalence of premarital individuals in the general population [8]

### Data used to fit the model

The model was fitted to syphilis prevalence data for high and low-risk FSWs and their clients at a single point in time. The syphilis prevalence data for FSWs came from the same study in Kaiyuan City which many of the behavioural parameters were drawn from [4], whereas a wide range was used for clients, taking the minimum lower bound and maximum upper bound from studies elsewhere in China [32], [38].

### Model fitting and impact projections

Combinations of biological and behavioural parameters were sampled 50,000 times from their uncertainty bounds using Latin hypercube sampling [39], assuming uniform parameter distributions. Any model simulations that projected an endemic syphilis prevalence within the FSW and client prevalence ranges were retained as model fits.

The baseline model analysis was conducted using the model that incorporated heterogeneity in the FSW population and incoming syphilis infection, but did not include regular non-commercial partners of FSWs (model 3). All fits for this model were used to estimate the 5-year impact of screening FSWs for syphilis on average every 6 months, 1, 2 or 4 years, using a rapid test with 87% sensitivity (field sensitivity for Standard Bioline test in China [15]), assuming that all positive FSWs receive immediate, effective treatment. The intervention's impact was estimated in terms of the relative change in syphilis prevalence and proportion of infections averted during and after the intervention programme. The full range of model fits were used to assess the uncertainty (95% central range) around the impact projections. The main drivers of uncertainty in the projected impact at the end of the intervention were determined using ANCOVA [40]. The outcome variable was the relative change in prevalence or number of infections averted amongst FSWs or clients at the end of the intervention, and all model parameters were entered simultaneously as explanatory variables, to identify which parameters accounted for >5% of the total sum of squares explained by all parameters.

The degree to which syphilis prevalence in FSWs rebounded above pre-intervention levels after the intervention ceased was investigated, and the rebound in infectious syphilis was also investigated for comparison with previous studies [18], [28]. The proportion of treatments which were correctly administered and the number of infections averted per FSW correctly treated were estimated over the course of the intervention, for two testing intervals (6 months and 4 years).

The fits for the other model structures (models 1, 2 and 4) were used to compare how the projected impact of yearly testing of all FSWs varied depending on the different model structural assumptions made, and to investigate their effect on the syphilis rebound and overtreatment. Overtreatment was assessed by keeping separate tallies of all those currently infected, and all those previously infected (immune or re-susceptible), who would test positive during the intervention (calculated per week as the product of the testing coverage, test sensitivity and total number of FSWs in that state), and comparing these numbers. The test was assumed to have 100% specificity amongst those never infected, so no overtreatment was calculated for the susceptible (S) group.

All results are reported as the median with the 95% central range in brackets.

## Results

### Impact projections for baseline model

**Figure 2:**
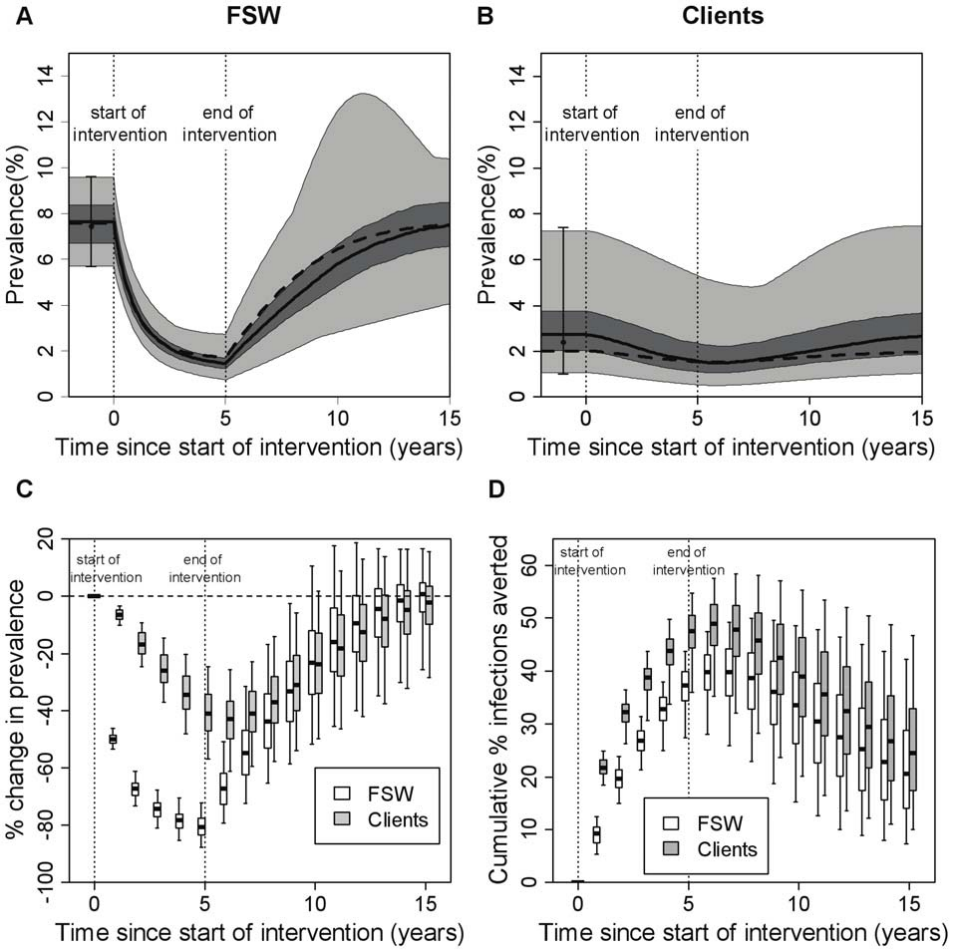
Projected intervention impact for the baseline model. The baseline model is model 3, which has a heterogeneous FSW population, incoming syphilis infection and no regular partners. A test with sensitivity of 87% was used, with FSWs being tested on average once per year, and with immediate treatment of all individuals testing positive. Results are shown over 5 years of the intervention and for an additional 10 years after the intervention stopped. Panels (**A**) and (**B**) summarise the range of prevalences seen across the different fits (N = 326) for (**A**) FSWs and (**B**) clients. The thick solid line shows the median, the dark shaded area shows the interquartile range (25^th^–75^th^ percentile), the light shaded area the full range (minimum-maximum), and the dashed line shows the best fit. The black circles with error bars represent the data (mean and range) that the model was fit to. In panels (**C**) and (**D**) impact is presented as (**C**) percentage change in prevalence (compared to pre-intervention endemic levels) and (**D**) percentage infections averted since the start of the intervention (compared with the situation where there was no intervention), and these are shown at yearly intervals. The thick horizontal line in each box is the median, with the box limits denoting the 25^th^ and 75^th^ percentiles and the whiskers denoting the 2.5^th^ and 97.5^th^ percentiles. The dotted vertical lines mark the start and end of the intervention.

For the baseline model (model 3, with FSW heterogeneity and incoming syphilis infections amongst new FSWs and clients), 326 out of 50,000 model simulations fit the FSW and client syphilis prevalence data ranges (see Text S1 for a description of the posterior parameter ranges and correlations between posteriors). For these model fits, Figure 2 shows the projected change in syphilis prevalence (both absolute-Figure 2A, B-and relative-Figure 2C) and infections averted (Figure 2D) during and after an intervention that implements yearly testing of all FSWs for 5 years. FSW syphilis prevalence declines by 81% (72–88%) after 5 years (Figure 2C). Client prevalence declines less, but continues to decrease for ∼1 year after the intervention ceases (Figure 2C). The cumulative number of syphilis cases averted increases during and for 5 years after the intervention ceases (data not shown), with a greater proportion of infections averted among clients than FSWs (Figure 2D). Most uncertainty in the impact projections is due to uncertainty in syphilis prevalence amongst new FSWs and clients, background treatment rates and the average duration of being a FSW or client (Text S1). Less impact is achieved for a higher prevalence amongst incoming FSWs/clients, higher background treatment of FSWs, lower background treatment of clients, and shorter durations of being a FSW or client.

**Figure 3:**
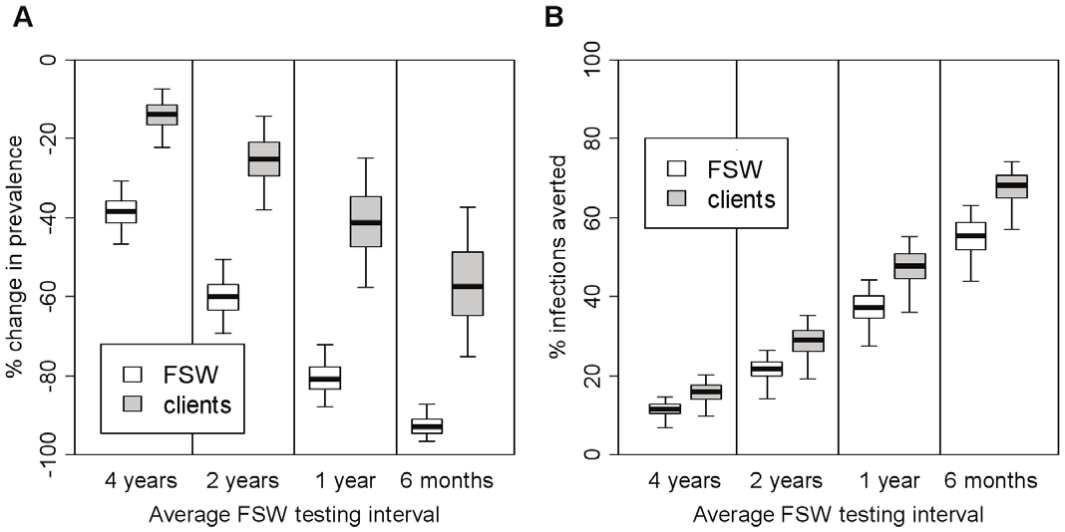
Effect of screening coverage on intervention impact after 5 years of intervention. Results are shown for both FSWs and their clients for all of the fits (N = 326) for model 3 (heterogeneous FSW population, incoming syphilis infection, no regular partners). A test with sensitivity of 87% was used, with immediate treatment of all individuals testing positive. Impact is presented as (**A**) relative reduction in prevalence (compared to pre-intervention levels) and (**B**) percentage infections averted since the start of the intervention (compared with the situation where there was no intervention). The thick horizontal line in each box is the median, with the box limits denoting the 25^th^ and 75^th^ percentiles and the whiskers denoting the 2.5^th^ and 97.5^th^ percentiles.

Higher testing frequencies increase the impact of screening (Figure 3), with an average 6 month or 4 year testing interval resulting in a 93% (87–97%) or 38% (31–47%) reduction in FSW syphilis prevalence after five years, respectively. Increasing test sensitivity has a similar effect. For example, if the test sensitivity is 95% (highest sensitivity achieved under laboratory conditions in China [15]), or 64% (lowest sensitivity achieved in a clinic setting in China [15]) then the 5-year impact of yearly testing is an 83% or 72% reduction in FSW syphilis prevalence, respectively.

### Rebound following intervention

**Figure 4:**
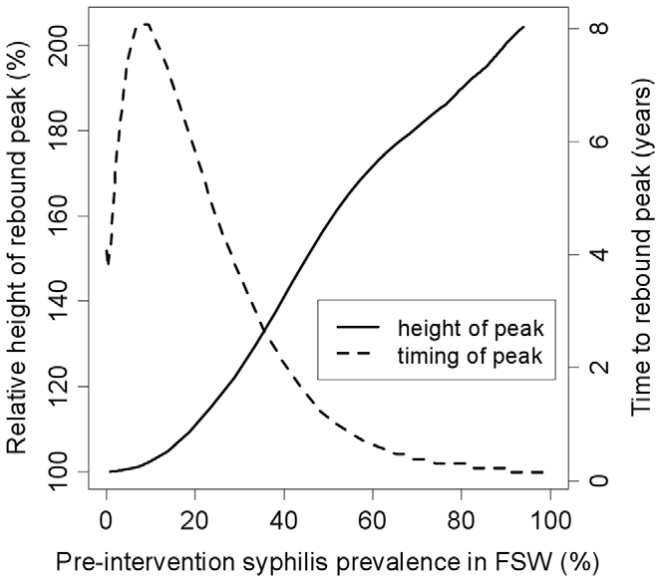
Timing and height of peak rebound in infectious syphilis for different FSW syphilis prevalence levels. Results are shown for model 3 using the best fit parameter set and varying 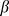 (transmission probability per act) between 0.01 and 1 to produce different epidemic settings. A 5-year intervention with yearly testing of all FSWs, using a rapid test of 87% sensitivity, was simulated. The x-axis shows overall pre-intervention syphilis prevalence (all infected stages) in the FSW population (high risk+low risk), and the rebound statistics shown are for infectious syphilis (primary, secondary and recurrent secondary stages) in the total FSW population (high risk+low risk).

After the intervention ceases, most baseline model simulations (324 of 326 fits for model 3) suggest that the FSW syphilis prevalence will temporarily rebound above pre-intervention levels. However, the rebound is likely to be slow and slight in this setting, with FSW prevalence peaking 13 years (7–36 years) after the intervention ceases, at a level 3.6% (0.002–20.5%) higher than pre-intervention prevalence. The rebound in infectious (primary/secondary stage) syphilis is slightly greater (5.8% (0.006–28.6%) above pre-intervention levels) and occurs about 3 years earlier. Since these rebounds were much slower and smaller than those reported in previous modelling analyses [18], [28], we undertook further analyses to understand the causes for this difference. Model projections suggest that much faster and more pronounced rebounds in syphilis prevalence (Figure 4) are likely to occur in higher syphilis prevalence settings (different syphilis prevalences obtained by varying transmission parameter 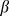
in the best fit for model 3). For example, if the FSW syphilis prevalence is >40% then the rebound in prevalence of infectious syphilis could occur within 2 years of the intervention ceasing and be over 40% greater than baseline.

### Overtreatment

**Figure 5:**
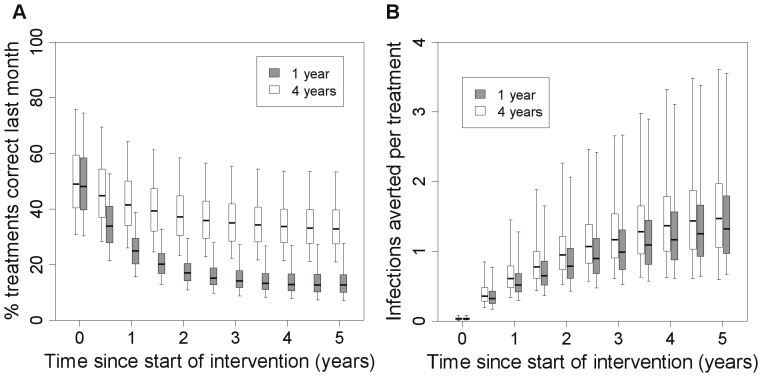
Overtreatment and treatment efficiency in model 3 at different intervention coverage. (**A**)% of treatments administered over the last month which were correct (i.e.% of those treated that have current infection rather than previous infection) over the course of a 5-year intervention using a rapid test with 87% sensitivity with a testing interval of 1 or 4 years as indicated, using model structure 3 (heterogeneous FSW population, incoming syphilis infection, no regular partners), (**B**) efficiency of treatment (total number of infections averted in FSWs and clients per treatment administered) over the last month, over this same period.

At the start of the intervention, a large proportion (50%) of treatments are given to uninfected FSWs because the rapid test does not distinguish between past and current infection. This proportion increases over the intervention (Figure 5A); after 5 years of yearly testing, 87% of treatments are given to uninfected FSWs. However, the total treatments per month decreases over the intervention (Figure S2) and the number of infections averted per treatment administered increases (Figure 5B). More overtreatment occurs with shorter testing intervals (Figure 5A).

### Effect of heterogeneity, incoming syphilis infections and re-infection from regular partners

**Figure 6:**
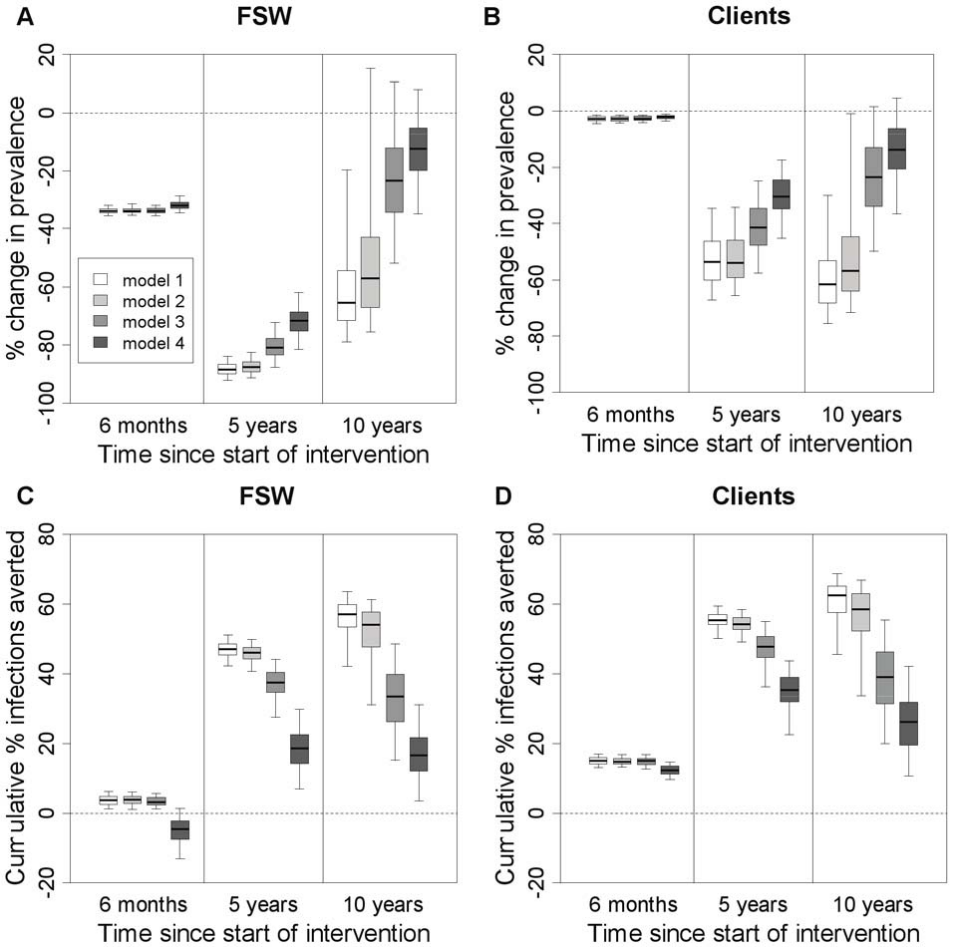
Impact of intervention on prevalence and percentage of infections averted for different model structures. Impact is shown at three different time points for FSWs (**A,C**) and clients (**B,D**). Impact is presented as relative change in prevalence (compared to pre-intervention levels) (**A,B**) and percentage infections averted since the start of the intervention (compared with the situation where there was no intervention) (**C,D**). Simulated intervention assumed FSW were screened once per year with a rapid test of 87% sensitivity, with all individuals testing positive receiving immediate treatment. The thick horizontal line in each box is the median, with the box limits denoting the 25^th^ and 75^th^ percentiles and the whiskers denoting the 2.5^th^ and 97.5^th^ percentiles. Impact is shown at 6 months, 5 years and 10 years after the start of a 5-year intervention (so that 10 years is 5 years after the end of the intervention). The different population models are: (**1**) baseline-homogeneous FSW population with no syphilis infection among FSWs and clients; (**2**) heterogeneous FSW population with no infection in new FSWs and clients; (**3**) heterogeneous FSW population with syphilis infection in both new FSWs and new clients; (**4**) heterogeneous FSW population with incoming syphilis infection and regular partners of FSW included.

For each model structure, Figure 6 shows the impact of yearly screening at three time points-6 months into the intervention, at the end of a 5-year intervention and 5 years after the intervention stops. After 6 months there is little difference between the models in terms of impact on prevalence, but differences emerge at later time points (Figure 6A,B). The inclusion of FSW heterogeneity in the model (model 2 vs. model 1) makes little difference to the predicted impact on prevalence, but incorporating incoming syphilis infections amongst new FSWs and clients (model 3 vs. model 2) leads to a reduction in the predicted impact on FSW prevalence −7% lower after 5 years of screening and 60% lower 5 years after the intervention ceases (Figure 6A). Incorporating re-infection from regular non-commercial partners of FSWs (model 4 vs. model 3) leads to even less impact on FSW prevalence at both time points (relatively 11% and 47% less at 5 and 10 years, Figure 6A).

The effect of model structure on infections averted is more pronounced. The model incorporating re-infection from regular non-commercial partners (model 4) projects less than half the infections averted among FSWs after 5 years than models which do not incorporate these partners (models 1–3) (Figure 6C). Among clients, this is less than 75% (Figure 6D). These differences are greater 5 years after the intervention ends, with the models which do not incorporate incoming syphilis infection (models 1 & 2) suggesting additional infections being averted after the intervention ceased, whereas models that do incorporate incoming syphilis infection (models 3 & 4) suggest decreases in infections averted 5 years after the intervention ceased (Figure 6C,D). Over the first 6 months, the model incorporating re-infection from regular non-commercial partners (model 4) suggests the intervention could result in a short-term increase in new FSW infections (Figure 6C). If the intervention is stopped at this point, a net benefit still occurs but takes 6 months to become apparent amongst FSWs (Figure S3). Little difference is seen in terms of infections averted when FSW heterogeneity is included in the model (model 2 vs. model 1), but a substantial decline in the proportion of infections averted is seen when incoming syphilis infections are included in the model (model 3 vs. model 2).

The rebound in syphilis prevalence occurs earlier but is less pronounced in the more complex models. The model without FSW heterogeneity, incoming infections or regular partners of FSW (model 1) suggests that FSW syphilis prevalence peaks on average 22 years after the intervention ends, at a prevalence 6.7% (0.08–37.3%) higher than pre-intervention levels. In contrast, the peak is only 2.5% (0.03–15.6%) higher for the model which includes all of these factors (model 4) and occurs 12 years after the intervention finishes. Model structure makes little difference to the projected level of overtreatment (results not shown).

## Discussion

Our analysis suggests that using rapid tests to screen FSWs for syphilis could have a large impact upon syphilis prevalence amongst FSWs and their clients in China. Although our analysis suggests substantial syphilis infections would be averted, especially amongst clients, the projected proportion of infections averted is reduced considerably (by 60% for FSWs and 33% for clients) if many new FSWs and clients are already infected with syphilis or many recently treated FSWs become re-infected by their regular non-commercial sexual partners. This illustrates the importance of reducing re-infection from regular non-commercial partners and of understanding the degree to which both effects occur in a specific setting. It is important that models assessing the impact of STI treatment interventions incorporate both these factors.

In contrast to previous modelling studies suggesting rapid and substantial rebounds in the prevalence of infectious syphilis could occur following mass syphilis treatment [18], [28], our analysis projected much slower and smaller rebounds in this FSW population. The faster and more pronounced rebounds predicted in previous studies were probably due to their models incorporating small core groups with very high syphilis prevalence (65–84% [18] and 38% [28]). This is consistent with our finding that faster and more pronounced rebounds are likely in settings with higher baseline syphilis prevalence.

One weakness of current rapid tests is that they cannot distinguish between past and current infections. Although our projections suggest this is likely to result in considerable (>50%) overtreatment, overall costs are unlikely to be greatly affected since the costs of testing the FSW population are much greater than the subsequent treatment costs for the smaller number testing positive [41]. Newly available rapid duplex syphilis tests (detecting both treponemal and non-treponemal antibodies) could reduce this overtreatment, but their performance has not yet been widely evaluated and their cost is prohibitively high (5–6 times greater than current treponemal rapid tests). However, they could be useful for testing individuals that have already tested positive with the treponemal rapid test.

Incorporating behavioural heterogeneity amongst FSWs had little effect upon the impact of screening in this setting, due to the modest difference (1.5–2 fold) in sexual activity and prevalence between the two risk groups. However, considering re-infections from regular partners greatly reduced the predicted impact-indeed, the model suggested that a temporary increase in syphilis incidence may be observed shortly after the start of a syphilis screening intervention due to a large number of FSWs, whose infection had been cleared by treatment, being rapidly re-infected by their regular partners. The importance of incorporating syphilis re-infection of FSWs from their regular non-commercial partners is in agreement with recent modelling considering the effects of ongoing partnerships on the impact of chlamydia screening [27]. Theoretically, testing and treatment of FSWs' regular non-commercial partners could partially reverse the reduction in impact attributable to re-infection. However, the feasibility of this strategy is uncertain– we are not aware of any previous efforts to trace and treat regular partners of FSWs for STIs, and few FSWs report that they would notify their sexual partners when they test positive for syphilis [42]. An alternative strategy might be to promote condom use by FSWs with their regular non-commercial partners, although it is not certain how high a level of condom use could be achieved, nor precisely how well condoms protect against syphilis transmission.

This analysis was limited by a lack of setting-specific data on the behaviour and syphilis prevalence of both clients and non-commercial partners, and on levels of existing treatment. Wide parameter ranges were used to account for uncertainties in the client and treatment data. However, point estimates were used for the non-commercial partner behaviour and so the results from model 4 should be seen as illustrative. The model did not consider new FSWs who had previously been cured of syphilis, which may affect estimates of overtreatment but not impact. All FSWs were also assumed to be equally likely to access the rapid test intervention, which may not be true. If high-risk FSWs are less likely to be screened then this may reduce impact. Lastly, the assumption that all FSWs testing positive receive immediate treatment is optimistic, meaning that impact may be overstated. This strategy differs from current Chinese national guidelines, which recommend referral to clinics for further testing and treatment.

In conclusion, point-of-care syphilis screening could dramatically decrease the prevalence of syphilis amongst FSWs in a typical Chinese setting, whereas large and rapid rebounds in syphilis prevalence following breaks in screening are unlikely to be an issue except in very high prevalence settings or populations. However, the continued introduction of new syphilis infections through incoming FSWs and clients, and the re-infection of FSWs through their regular non-commercial partners can both considerably diminish the impact of FSW screening. The importance of these effects in a specific setting should be evaluated when planning STI screening strategies.

## Supporting Information

Figure S1
**Schematic diagram showing the different population groups/states within the pairwise model (model 4).** FSW and clients may be infected or uninfected, and FSW may be single or have a regular partner, and if they have a regular partner, that partner may be infected or uninfected. Squares = clients; circles = FSWs (top level), regular partners (bottom level). Possible movements between different states are shown by arrows. High and low FSW risk groups are not shown separately for clarity-rates of FSW infection by clients are shown as 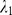 for high-risk FSWs, 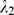
for low-risk FSWs, rates of screening and treatment 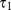 for high-risk FSWs, 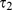
for high-risk FSWs and rates of background treatment 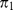 for high-risk FSWs, 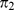
for low-risk FSWs.(TIF)

Figure S2 **Total treatments administered in model 3 at different testing intervals.** Total number of treatments administered over the course of a 5-year intervention using a rapid test with 87% sensitivity with a testing interval of 1 or 4 years as indicated, using model structure 3 (heterogeneous FSW population, incoming syphilis infection, no regular partners).
(TIF)

Figure S3 **Projected impact of a short intervention for model 4 (including regular partners).** Results are shown at yearly intervals from the end of a 6 month-long intervention (during which FSWs were tested on average once a year), for 10 years after the intervention stopped, for all of the fits (N = 398) for model 4. A test with sensitivity of 87% was used, with immediate treatment of all individuals testing positive. Impact is presented as (**A**) percentage change in prevalence (compared to pre-intervention levels) and (**B**) percentage infections averted since the start of the intervention (compared with the situation where there was no intervention). The thick horizontal line in each box is the median, with the box limits denoting the 25^th^ and 75^th^ percentiles and the whiskers denoting the 2.5^th^ and 97.5^th^ percentiles.
(TIF)

Text S1 Differential equations for syphilis transmission model, posterior parameter distributions, and uncertainty in impact projections.
(DOCX)
