# Supplementary figures and images for: The Impact of Syphilis Screening among Female Sex Workers in China: A Modelling Study
Source: PLoS One. 2013 Jan 30;8(1):e55622. doi: 10.1371/journal.pone.0055622 (PMC3559538; doi:10.1371/journal.pone.0055622)

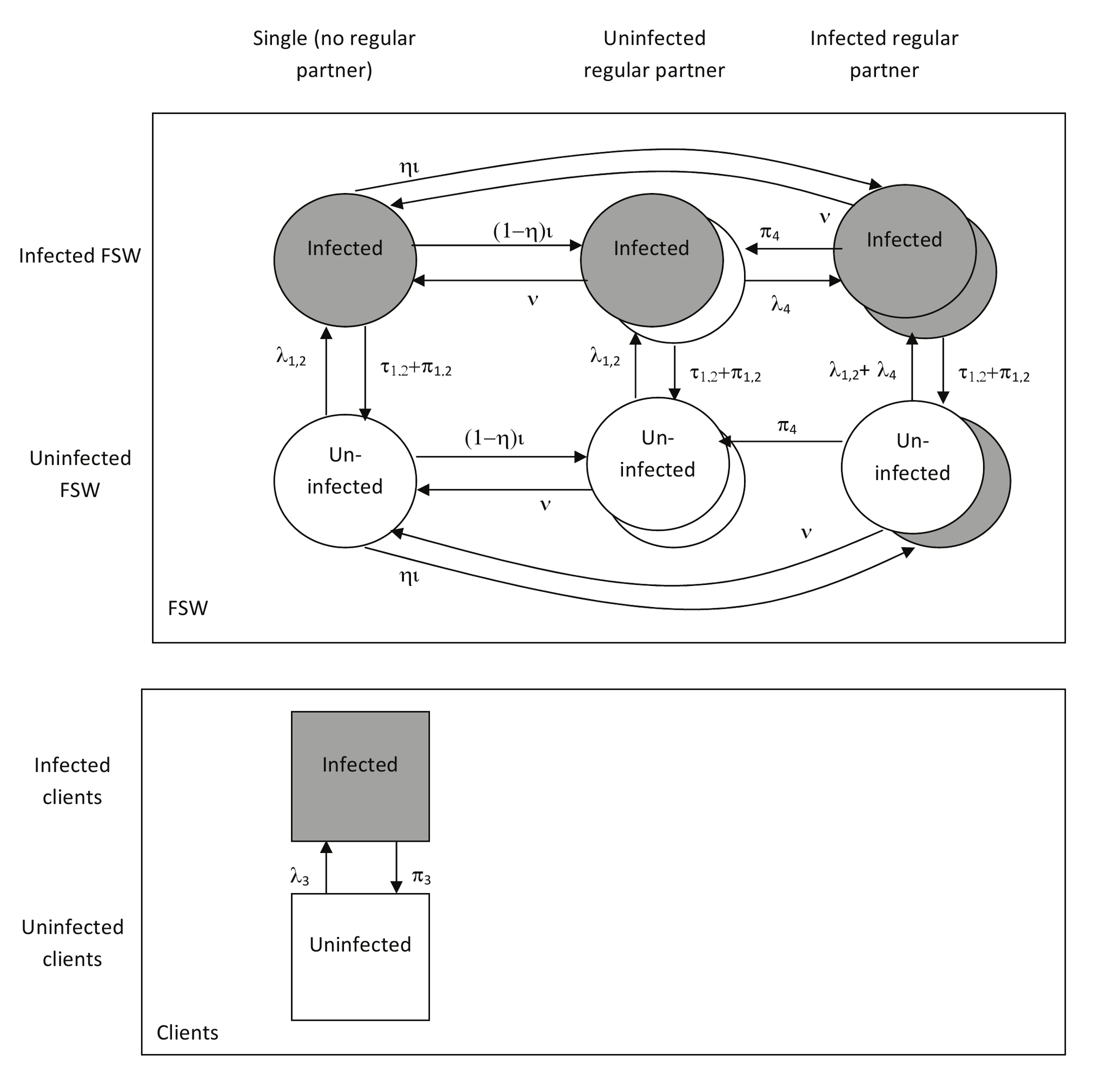

Supplement: Figure S1 — Schematic diagram showing the different population groups/states within the pairwise model (model 4). FSW and clients may be infected or uninfected, and FSW may be single or have a regular partner, and if they have a regular partner, that partner may be infected or uninfected. Squares = clients; circles = FSWs (top level), regular partners (bottom level). Possible movements between different states are shown by arrows. High and low FSW risk groups are not shown separately for clarity-rates of FSW infection by clients are shown as for high-risk FSWs, for low-risk FSWs, rates of screening and treatment for high-risk FSWs, for high-risk FSWs and rates of background treatment for high-risk FSWs, for low-risk FSWs. (TIF) [file pone.0055622.s001.tif]

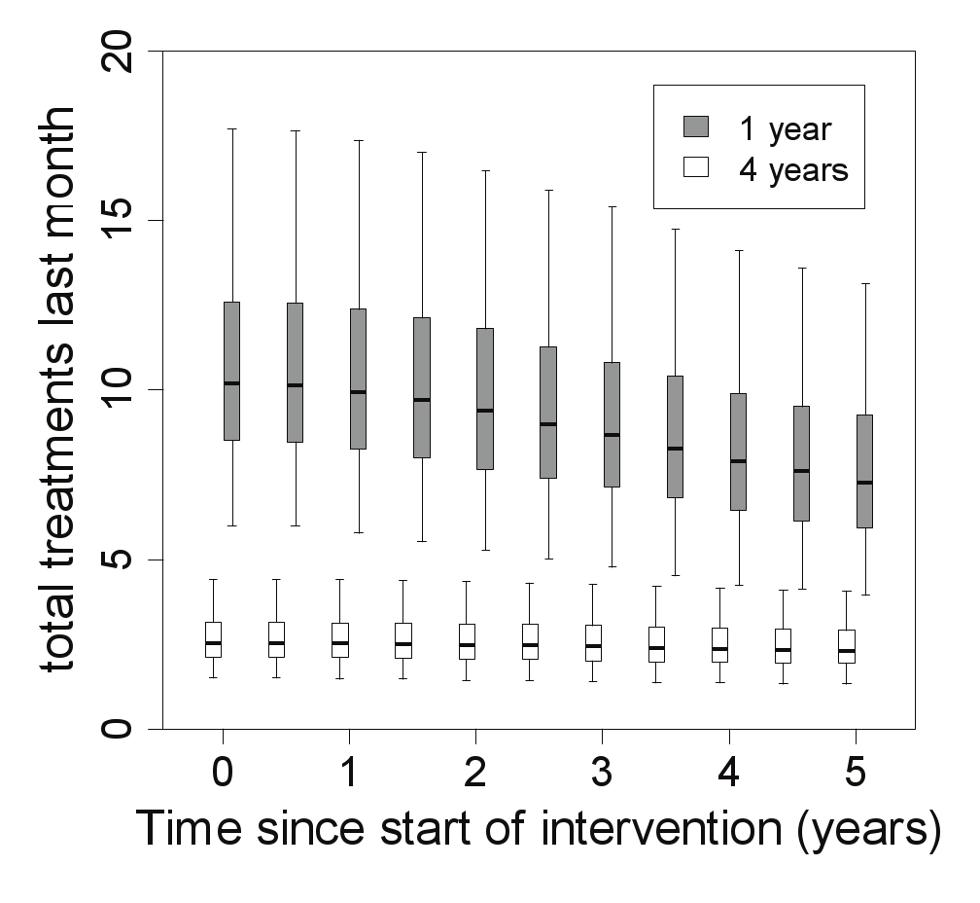

Supplement: Figure S2 — Total treatments administered in model 3 at different testing intervals. Total number of treatments administered over the course of a 5-year intervention using a rapid test with 87% sensitivity with a testing interval of 1 or 4 years as indicated, using model structure 3 (heterogeneous FSW population, incoming syphilis infection, no regular partners). (TIF) [file pone.0055622.s002.tif]

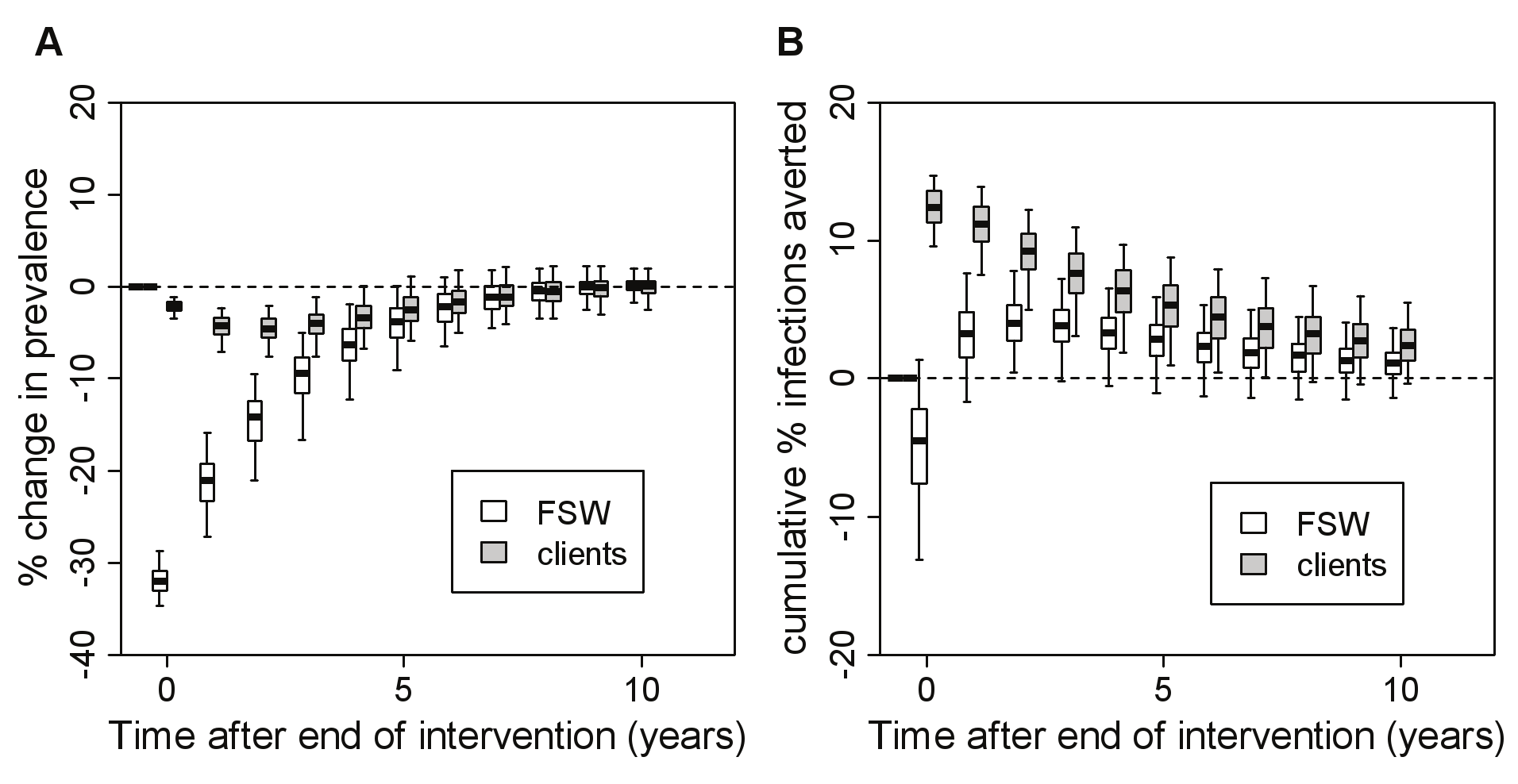

Supplement: Figure S3 — Projected impact of a short intervention for model 4 (including regular partners). Results are shown at yearly intervals from the end of a 6 month-long intervention (during which FSWs were tested on average once a year), for 10 years after the intervention stopped, for all of the fits (N = 398) for model 4. A test with sensitivity of 87% was used, with immediate treatment of all individuals testing positive. Impact is presented as (A) percentage change in prevalence (compared to pre-intervention levels) and (B) percentage infections averted since the start of the intervention (compared with the situation where there was no intervention). The thick horizontal line in each box is the median, with the box limits denoting the 25th and 75th percentiles and the whiskers denoting the 2.5th and 97.5th percentiles. (TIF) [file pone.0055622.s003.tif]
