# Supplementary material for: The Impact of Syphilis Screening among Female Sex Workers in China: A Modelling Study
Source: PLoS One. 2013 Jan 30;8(1):e55622. doi: 10.1371/journal.pone.0055622 (PMC3559538; doi:10.1371/journal.pone.0055622)
Supplement: Text S1 — Differential equations for syphilis transmission model, posterior parameter distributions, and uncertainty in impact projections. (DOCX) [file pone.0055622.s004.docx]

# Supporting Information Text S1

# Differential equations for syphilis transmission, diagnosis and treatment model

## Infection transition equations

The following equations describe the transitions between the different infection stages for FSWs and clients (omitting regular partners of FSWs).

|  | $\frac{dS_{j}}{dt}=\left( 1-\eta_{j} \right)\mu_{j}N_{j}-S_{j}\left( \lambda_{j}+\mu_{j} \right)$ | (1) |
| --- | --- | --- |
|  | $\frac{dI_{j,1}}{dt}=\lambda_{j}\left( S_{j}+Y_{j}+\left( 1-\gamma\right)R_{j} \right)-I_{j,1}\left( {\sigma_{1}+\tau}_{j}+ \pi_{j}+\mu_{j} \right)$ | (2) |
|  | $\frac{dI_{j,2}}{dt}=\sigma_{1}I_{j,1}-I_{j,2}\left( {\sigma_{2}+\tau}_{j}+ \pi_{j}+\mu_{j} \right)$ | (3) |
|  | $\frac{dI_{j,3}}{dt}=\eta_{j}\mu_{j}N_{j}+\sigma_{2}I_{j,2}-I_{j,3}\left( {\sigma_{3}+\tau}_{j}+ \pi_{j}+\mu_{j} \right)$ | (4) |
|  | $\frac{dI_{j,4}}{dt}={\phi\sigma}_{3}I_{j,3}-I_{j,4}\left( \sigma_{4}+\tau_{j}+ \pi_{j}+\mu_{j} \right)$ | (5) |
|  | $\frac{dI_{j,5}}{dt}=\sigma_{4}I_{j,4}-I_{j,5}\left( \sigma_{5}+\tau_{j}+ \pi_{j}+\mu_{j} \right)$ | (6) |
|  | $\frac{dI_{j,6}}{dt}={{\left( 1-\phi\right)\sigma}_{3}I_{j,3}+\sigma}_{5}I_{j,5}-I_{j,6}\left( \tau_{j}+ \pi_{j}+\mu_{j} \right)$ | (7) |
|  | $\frac{dR_{j}}{dt}=\left( \pi_{j}+\tau_{j} \right)\left( I_{j,4}+I_{j,5}+I_{j,6} \right)-R_{j}\left( \left( 1-\gamma\right)\lambda_{j}+\sigma_{R}+\mu_{j} \right)$ | (8) |
|  | $\frac{dY_{j}}{dt}=\left( \pi_{j}+\tau_{j} \right)\left( I_{j,1}+I_{j,2}+I_{j,3} \right)+\sigma_{R}R_{j}-Y_{j}\left( \lambda_{j}+\mu_{j} \right)$ | (9) |

The index j represents low-risk FSWs (j = 1), high-risk FSWs (j = 2) or clients (j = 3). S are susceptible individuals, I are infected, R are those with partial temporary immunity and Y are those previously exposed and susceptible (rapid test positive). The index k represents the stage of infection for the infected (I) states, from incubating (k=1) through primary (k=2), secondary (k=3), remission (k=4), recurrent secondary (k=5) and latent tertiary (k=6).

The treatment rate (τ_j_) when the intervention is in place is the product of the rate of screening of that group (α_j_), diagnostic test sensitivity (ω), and treatment efficacy (θ; assumed to be 100% in this analysis):

|  | $\tau_{j}=\alpha_{j}\omega\theta$ | (10) |
| --- | --- | --- |

For infections of FSWs by clients, or of clients by FSWs, the force of infection λ_j_ is given by:

|  | $\lambda_{j=1,2}=\left( 1-\zeta_{j}\rho\right)\beta C_{j}\frac{\left( I_{3,2}+I_{3,3}+\Delta I_{3,5} \right)}{N_{3}}$ | (11) |
| --- | --- | --- |
|  | $\lambda_{3}=\beta C_{3}\sum_{j=1}^{2} \left( \left( \frac{C_{j}N_{j}}{{C_{1}N_{1}+C}_{2}N_{2}} \right)\left( 1-\zeta_{j}\rho\right)\frac{\left( I_{j,2}+I_{j,3}+\Delta I_{j,5} \right)}{N_{j}} \right)$ | (12) |

where λ_1_ is the force of infection for low risk FSWs from their clients, λ_2_ is the force of infection for high risk FSWs from their clients and λ_3_ is the force of infection for clients from FSWs. Δ is the proportion infectious in the recurrent secondary stage I _j ,5_, ρ is the efficacy of condoms against syphilis transmission, ζ_j_ is the consistency of condom use (j = 1 for low-risk FSWs with clients, j = 2 for high-risk FSWs with clients), I_j,k_ is the number of infected FSWs or clients in risk group j and stage k, N_j_ is the total population size for group j and C_j_ is the total number of different commercial partners per individual in group j per time period. The assumed size of the client population is determined by the size and demand for partnerships of the FSW populations and by the number of partnerships each client forms on average. This is calculated as:

|  | $N_{3}=\frac{C_{1}N_{1}+C_{2}N_{2}}{C_{3}}$ | (13) |
| --- | --- | --- |

**Variables**

μ_j_ = Model entry and exit rate

σ_i_ = Rate of leaving syphilis infection stage i

Φ = Proportion entering remission stage from secondary infection, remainder go to latent tertiary

γ = Degree to which partial immune protection decreases susceptibility to re-infection

τ_j_ = Intervention treatment rate

λ_j_ = Force of infection for low-risk FSWs from clients (j=1), high-risk FSWs from clients (j=2), clients from FSWs (j=3), FSWs from regular partners/regular partners from FSWs (j=4)

ρ = Efficacy of condoms in protecting against transmission of syphilis

ζ_j_ = Consistency of use of condoms for low-risk FSWs with clients (j = 1), for high-risk FSWs with clients (j = 2) and for FSWs (either risk group) with their regular partners (j=4)

α_j_= Rate of screening for FSW group j

θ = Efficacy of treatment

ω = Test sensitivity

β = Syphilis transmission probability per commercial sex act

Δ = Proportion infectious within recurrent secondary stage

C_j_= Number of commercial sex partners per time period for low risk FSWs (j=1), high risk FSWs (j=2) and clients (j=3)

N_j_ = Number in population in FSW/client group j

π_j_ = Background rate of treatment for individuals in group j

**FSW / Client Indices (j)**

1= FSWs low risk

2= FSWs high risk

3= Clients

**Infection Stage Indices (k)**

1 = Incubating

2 = Primary

3 = Secondary

4 = Remission

5 = Recurrent Secondary

6 = Latent / Tertiary

## Pairwise model including regular partners of FSWs: model description

Regular partners of FSWs are included in model 4 using pairwise partnership modelling^1, 2^, with FSWs stratified into single or partnered states. These FSW groups are then stratified further into infected (all stages) and uninfected states, with infected or uninfected partners. These groups, and all of the possible transitions between them, are shown schematically in figure S1. Single FSWs form long-term partnerships at a per-capita rate of ι per week, with a fraction (η_j_) of these new regular male partners already being infected with syphilis (these are assumed to be in the secondary stage of infection), and the remainder (1-η_j_) being uninfected. Regular partnerships dissolve at a rate of ν per week, with FSWs returning to the single state, and their male partners leaving the population. Uninfected individuals become infected at a rate λ_j_, where λ_1_(λ_2_) is the rate at which low-risk (high-risk) FSWs are infected by their clients, λ_3_ is the rate at which clients are infected by FSWs, λ_4_ is the rate at which regular partners and FSWs infect each other. Infected regular partners return to the uninfected state due to background treatment, at a per-capita rate π_4_. A fraction (χ) of new FSWs are assumed to be single, with the remainder (1-χ) having a regular partner, of whom a fraction (η_j_) are assumed to be already infected with syphilis. The model equations are presented in the next section (A.3).

## Pairwise partnership transition equations

The following equations describe the transitions for FSWs between different partnership states for model 4, including movements between the infected and uninfected states, and between having an infected and uninfected regular partner.

| $\frac{dU_{0j}}{dt}=\chi_{j}\left( 1-\eta_{j} \right)\mu_{j}N_{j}+V_{0j}\left( \tau_{j}+\pi_{j} \right)+\nu\left( U_{1j}+U_{2j} \right)-U_{0j}\left( \lambda_{j}+\mu_{j}+\iota_{j} \right)$ | (14) |
| --- | --- |
| $\frac{dU_{1j}}{dt}=\left( 1-\chi_{j} \right)\left( 1-\eta_{j} \right)\mu_{j}N_{j}\left( 1-\eta_{4} \right)+V_{1j}\left( \tau_{j}+\pi_{j} \right)+\left( 1-\eta_{4} \right)\iota_{j}U_{0j}+\pi_{4}U_{2j}-U_{1j}\left( \lambda_{j}+\mu_{j}+\nu_{j} \right)$ | (15) |
| $\frac{dU_{2j}}{dt}=\left( 1-\chi_{j} \right)\left( 1-\eta_{j} \right)\mu_{j}N_{j}\eta_{4}+V_{2j}\left( \tau_{j}+\pi_{j} \right)+\eta_{4}\iota_{j}U_{0j}-U_{2j}\left( \lambda_{j}+{\lambda_{4}+\pi}_{4}+\mu_{j}+\nu_{j} \right)$ | (16) |
| $\frac{dV_{0j}}{dt}=\chi_{j}\eta_{j}\mu_{j}N_{j}+{\lambda_{j}U}_{0j}+\nu\left( V_{1j}+V_{2j} \right)-V_{0j}\left( \tau_{j}+\pi_{j}+\mu_{j}+\iota_{j} \right)$ | (17) |
| $\frac{dV_{1j}}{dt}=\left( {1-\chi}_{j} \right)\eta_{j}\mu_{j}N_{j}\left( 1-\eta_{4} \right)+{\lambda_{j}U}_{1j}+\left( 1-\eta_{4} \right)\iota_{j}V_{0j}+\pi_{4}V_{2j}-V_{1j}\left( \tau_{j}+\pi_{j}+\mu_{j}+\nu_{j}+\lambda_{4} \right)$ | (18) |
| $\frac{dV_{2j}}{dt}=\left( {1-\chi}_{j} \right)\eta_{j}\mu_{j}N_{j}\eta_{4}+{\left( \lambda_{j}+\lambda_{4} \right)U}_{2j}+\eta_{4}\iota_{j}V_{0j}+\lambda_{4}V_{1j}-V_{2j}\left( \tau_{j}+\pi_{j}{+ \pi}_{4}+\mu_{j}+\nu_{j} \right)$ | (19) |

where U is uninfected FSWs (S+R+Y) and V is infected FSWs (I_1_+I_2_+I_3_+I_4_+I_5_+I_6_). The first subscript for these states indicates partner status: 0 = no regular partner, 1 = have an uninfected regular partner, 2 = have an infected regular partner. The second subscript (j) indicates high or low risk FSW group. For clarity, these equations over-simplify the infection process: in reality, only individuals in the infectious stages can transmit infection. The equations for the force of infection between FSWs and clients are the same in the pairwise model as in the model without regular partners (equations 11 and 12). In the regular partner model, infection may additionally be transmitted between FSWs and their regular partners, whenever one of them is susceptible to infection and the other is in an infectious state. The force of infection (in either direction) if one of the pair is in the S or Y stage and the other is in the I_2_ or I_3_ stage is:

|  | $\lambda_{4}=\left( 1-\zeta_{4}\rho\right)\beta C_{4}$ | (20) |
| --- | --- | --- |

where λ_4_ is the force of infection for regular partners from FSWs or for FSWs from regular partners, ζ_4_ is the consistency of condom use for FSWs with regular partners and C_4_ is the coital frequency within regular partnerships. If the susceptible partner is in the R stage, λ_4_ is multiplied by (1-γ), and if the infectious partner is in the I_5_ stage λ_4_ is multiplied by Δ.

The full model equations for model 4 incorporate the 9 different susceptible/infection states in equations 1-9 for FSWs, clients and regular partners within the pairwise partnership framework described in equations 14-19.

# Posterior parameter distributions

For all of the models, it was seen that there was little restriction of the range of any of the behavioural or biological parameters, but the posterior distributions for several parameters differed considerably from the prior distribution. Lower values for the duration of the secondary phase of infection (1/σ_3_), the transmission probability per partnership (β) and the client contact rate (C_3_) were generally seen in fits (relative to prior values) in all of the models. Higher levels of ρ (condom efficacy) were seen in fits (relative to prior values) in all of the models. In model 2 – heterogeneous FSW population with no prior infection in those entering the FSW or client populations - higher levels of γ (degree of partial immune protection) were seen relative to prior values, and in all of the models with heterogeneity within the FSW population (models 2, 3 and 4), contact rates for high-risk FSWs (C_2_) tended to be lower than the input values.

Strong correlations were observed between some of the posterior distributions for different parameters. A strong negative correlation between the duration of the secondary phase of infection (1/σ_3_) and the transmission probability per partnership (β) was consistently seen in all models. Additionally, a positive correlation between the transmission probability per partnership (β) and condom efficacy against syphilis (ρ) was seen for all models (most strongly in models 1 and 2). In the models with heterogeneity within the FSW population (models 2, 3 and 4), a negative correlation was seen between the transmission probability (β) and contact rates for high-risk FSWs (C_2_), and a positive association was seen between the contact rates of high- and low-risk FSWs (C_1_ and C_2_). This latter association is suggestive of some limit on the maximum ratio between contact rates for these two groups required to give fits to syphilis prevalence in both groups.

# Uncertainty in impact projections

At the end of a 5-year intervention, uncertainty in the projected relative change in syphilis prevalence amongst FSWs is due to uncertainty in a number of parameters, including the prevalence of syphilis among new FSWs and clients, which accounts for 39% of the total sum of squares accounted for by all of the model parameters, background treatment rate amongst FSWs (accounting for 20% of the variability), and the average duration that high risk FSWs, low risk FSWs and clients are involved in commercial sex (13%, 12% and 9% respectively). Increases in the prevalence of incoming syphilis infection will reduce the intervention’s impact in reducing syphilis prevalence due to the higher number of infected individuals entering the population during the intervention. Increases in the background level of treatment amongst FSWs, and decreases in the duration spent as a FSW/client all tend to decrease the intervention’s impact in decreasing syphilis prevalence because these model fits need to have higher syphilis incidence to achieve the same prevalence. Similarly, variability in the relative change in syphilis prevalence amongst clients is mostly due to uncertainty in the background level of treatment amongst clients, with it accounting for 70% of the variability, with the prevalence of syphilis among new FSWs and clients accounting for a further 21% of the variability. Increases in the background level of client treatment increase the intervention’s impact on client prevalence because any decreases in syphilis incidence amongst the clients becomes more quickly translated into reductions in prevalence. As for FSWs, increases in the prevalence of incoming syphilis infection will reduce the intervention’s impact in reducing syphilis prevalence due to the higher number of infected individuals entering the population during the intervention.

Variability in the projected proportion of infections averted amongst FSWs after 5 years is largely due to uncertainty in four parameters: the prevalence of syphilis among new FSWs and clients (which accounts for 56% of variability), the average duration for which clients are involved in commercial sex (accounting for 18% of variability), the degree of partial immune protection (6%) and the number of commercial partners that high-risk FSWs have per month (5%). Increases in the prevalence of incoming infection and the contact rate for high-risk FSWs both tend to decrease the proportion of new infections averted, while increases in the degree of partial immune protection and duration of being a client tend to increase the proportion of new infections averted. Lastly, variability in the projected proportion of infections averted amongst clients is mainly due to uncertainty in the prevalence of syphilis among new FSWs and clients, the average duration for which clients are involved in commercial sex, the syphilis transmission probability, commercial contact rate of high-risk FSWs, the degree of partial immune protection and the duration of the secondary syphilis stage which account for 53%, 10%, 6%, 6%, 5% and 5% of the uncertainty in the impact projections, respectively. Increases in the prevalence of incoming infection, the transmission probability and the contact rate for high-risk FSWs tend to decrease the number of infections averted amongst the clients whereas increases in the duration of secondary syphilis, the degree of partial immune protection and the duration of being a client all increase the number of infections averted.

**References**

1. Ferguson NM, Garnett GP. More realistic models of sexually transmitted disease transmission dynamics - Sexual partnership networks, pair models, and moment closure. *Sex Transm Dis* 2000;**27**: 600-9.

2. Kretzschmar M, Dietz K. The effect of pair formation and variable infectivity on the spread of an infection without recovery. *Math Biosci* 1998;**148**: 83-113.
